# The long-term effect of the coronavirus pandemic on parkrun participation: an interrupted time series analysis

**DOI:** 10.1186/s12889-024-20420-0

**Published:** 2024-10-23

**Authors:** Oscar Rousham, Helen Quirk, Elizabeth Goyder, Robert A. Smith

**Affiliations:** Sheffield Centre for Health and Related Research (SCHARR), The University of Sheffield, Sheffield, S10 2TN UK

**Keywords:** Parkrun, Physical activity, Socioeconomic deprivation, Ecological study, Interrupted time series

## Abstract

**Background:**

The growth of parkrun between 2004 and 2019 has been heralded as a success story for public health as a result of its physical activity and wellbeing benefits for participants. However, parkrun was not immune from the COVID-19 pandemic - with events in mainland England cancelled from March 2020 to July 2021. This study explores the lasting impact of the pandemic on parkrun participation to February 2023, and its implications across the socioeconomic spectrum.

**Methods:**

The study combines aggregated parkrun weekly finisher data from 32,470 Lower Layer Super Output Areas (LSOA) in England from January 2015 to February 2023 with Office of National Statistics (ONS) data on population and deprivation. Interrupted time series analysis using segmented Poisson regression models was used to estimate the immediate change in parkrun participation and the change in the rate of growth following the pandemic. Models were fitted for each Index of Multiple Deprivation (IMD) quintile separately to assess whether this effect differed by socioeconomic deprivation.

**Results:**

Visualisation and interrupted time series analysis showed a significant and long-term decrease in parkrun participation following the reopening of parkrun events. This was consistent across all IMD quintiles, indicating that the inequalities in parkrun participation according to IMD observed prior to the pandemic remained after the pandemic. Between March 2020 and February 2023, almost 13 million fewer parkrun finishes are estimated to have occurred relative to what would have occurred in the absence of the pandemic.

**Conclusion:**

The reduction in parkrun participation during the pandemic and following the reopening of events is likely to have negatively impacted wellbeing in would-be participants. Going forwards, policymakers must make the difficult trade-off between the long-term health and social implications of restricting outdoor physical activity events against the benefits associated with a reduction in infectious disease transmission.

## Introduction

Engaging in regular physical activity is linked to a decreased risk of developing numerous non-communicable diseases [11], along with notable reductions in depression and anxiety [31], as well as an increase in overall productivity [9]. However, a significant portion of the population falls short of recommended activity levels [13] and there is socioeconomic inequality in leisure time physical activity levels [1, 3, 19]. Elevating population-wide physical activity has the potential to improve public health and wellbeing and to reduce healthcare pressures and expenditure [17].

parkrun (written with a lowercase ‘p’) is a charity which organises and supports volunteer-delivered weekly 5 kilometre running and walking events (parkruns) in local communities. parkrun is widely considered to be a successful public health intervention (e.g. [33]), with over 750 events in the UK and a weekly attendance in the hundreds of thousands, many of whom were not physically active prior to participating in parkrun. Participants who complete the 5 kilometre course are given a token at the finish-line, which they are invited to scan, along with their unique parkrun ID number (barcode), to link their result to their personal parkrun profile. The vast majority do so. This allows participants to keep a record of all of the events they have participated in, but also provides parkrun with a rich dataset of the 9 million parkrun registrants worldwide.

parkrun participation is associated with improved self-reported fitness and mental wellbeing [16, 26] and brings improvements in social capital to the hosting community via volunteers and networks formed around parkrun [32]. parkrun has the capacity to improve physical activity levels in participants, particularly as many did not meet current physical activity guidelines prior to participating in parkrun [4, 15].

parkrun also has the potential to reduce the inequalities in physical activity present in England [23] by promoting physical activity in deprived groups [15], which is a key aim of Sport England’s ‘Uniting the Movement’ strategy [24]. However, previous research has shown that more socioeconomically deprived communities had lower parkrun participation rates than less deprived communities, despite living closer on average to events [20, 22]. Increasing parkrun participation in lower socio-economic groups is a central aim of parkrun, and geospatial analysis has helped to inform the locations of new events in order to achieve more equitable access and to help improve participation in more deprived communities [18, 21].

The coronavirus pandemic had wide ranging effects on general physical activity levels [27]. Studies suggest that these effects continued beyond the pandemic period [23, 29] and particularly negatively impacted those of lower socioeconomic position which may lead to a lasting increase in socioeconomic inequality in physical activity levels [5, 23].

parkrun was profoundly impacted by the pandemic. All parkrun events in mainland England were paused from 18th March 2020 until 24th July 2021 when events began to reopen in accordance with the UK Government’s roadmap out of lockdown [30]. Initial high-level data from parkrun suggests that weekly participation in the months following the return of parkrun was substantially lower than before the pandemic. Further, a study looking at specific parkrun events in Scotland showed that attendance in the year after the return to parkrun was 13% lower than the year before the pandemic [7].

Based on this evidence, we hypothesise that the hiatus in parkrun events may have had a negative effect on parkrun participation and, as observed for other physical activity [5, 23], that this impact may be larger in communities of lower socioeconomic position. To test this hypothesis, we use interrupted time series analysis to quantify the immediate impact and change in growth of parkrun participation following the pandemic controlling for the pre-pandemic trend in participation and confounding factors such as seasonality. We go on to quantify the loss in the number of parkrun finishers associated with the pandemic for the period from March 2020 to February 2023 by comparing observed data to estimated participation in the absence of the pandemic.

The results of this study will provide an insight into the long-term effect of the pandemic on a successful mass participation community level physical activity event and may add to the body of evidence on the long-term implications of different courses of action in future pandemics. Importantly, this study considers the impact of the pandemic as a whole and does not aim to quantify the effects of specific policies, behavioural shifts or the response of parkrun UK.

## Methods

### Dataset and population

Data on weekly parkrun finishes for each of the 32,844 Lower Super Output Areas (LSOA) based on participant’s home address in England between January 2010 and February 2023 was provided to the research team by parkrun. LSOAs are small geographical areas, with a population of 1,000 to 3,000 people, defined by the Office for National Statistics for admistration and statistics. IMD scores (a composite measure of deprivation based on measures including average levels of employment, crime, education, and income) and population estimates for each LSOA were obtained from the English Indices of Deprivation 2019 [14] (Table 1).

Data on parkrun participation prior to 2015 was discarded because parkrun growth prior to 2015 had exponential growth distinct from the near-linear trend observed since 2015. Where historical trends differ from the current trend it is recommended that the former is not included in time series analysis [2]. Only parkruns occurring on a Saturday and in England were included in analysis. Junior parkruns and parkrun events taking place in custodial sites were excluded. The data only included runners and walkers who completed a parkrun event and scanned their parkrun barcode.

**Table 1:** Variables used in this study and their source

Variable	Description	Source
Weeks	Weeks since 1st January 2015	NA
finishers	Number of finishers per week	parkrun UK
imd_q5	Index of Multiple deprivation scores by LSOA	MHCLG, 2019
total_pop	Number of residents in each LSOA	MHCLG, 2019
participation_rate	Number of finishers per 1000 residents	derived

### Outcome

The outcome for this study was weekly parkrun participation rates. That is, the number of parkrun finishes per 1000 residents. Specifically, the immediate change (step-change) and change in slope of weekly parkrun participation rates following the reopening of parkrun events after the pandemic period.

### Exposures

The reopening of parkrun events took place on Saturday 24th July 2021. IMD deciles were linked to parkrun finisher data at the LSOA level based on each finisher’s home address and collapsed into quintiles. Population estimates and IMD scores were assumed to change little throughout the study period and therefore values from the most recent report, Indices of Deprivation 2019 [14], were used throughout.

### Covariates

Time was measured as weeks since 1st January 2015. Seasonality was categorised as a 13-level variable with each level representing a four-week period of the year. This accounts for predictable and recurring fluctuations in participation influenced by changes across the year, such as New Year Resolutions, the start of a new school year, and seasonal weather. The ‘pandemic period’ was defined as 21st March 2020 (the first week after parkrun events were paused) until 24th July 2021 (when parkrun events reopened).

### Analysis

Parkrun participation rates (finishes per 1000 residents) were stratified by IMD and visualised over time. The number of events held each week were plotted on the same graph. In order to estimate the impact of the pandemic on parkrun participation, we first defined three distinct periods: pre-pandemic, whilst events were paused, and after events resumed. An interrupted time series model was then fit first for the overall population and then for each IMD quintile separately. These models included two key parameters: First, a step-change which captures the difference between the weekly number of participants observed upon the return of parkrun events with the equivalent weekly number of participants expected had the pandemic not occurred. Second, the change in the growth of parkrun participation which compares the pre-pandemic trend in weekly participation to the post-pandemic trend. The pre-pandemic trend was extrapolated across the study period to establish a counterfactual. That is, the predicted weekly parkrun participation had the pandemic not occurred. The difference between the counterfactual and the observed finishes was calculated to quantify the total number of lost parkrun finishes between March 2020 and February 2023 associated with the pandemic. Interrupted time series was conducted using quasi-poisson regression due to the high variance in the number of weekly finishers. Visualisation of pre-pandemic trends of weekly participation suggested near-linear growth. However, this growth could not be expected to continue indefinitely and was expected to conform to an S-curve shape. As such, linear and quadratic terms for the change in participation over time were included in the model to allow the rate of growth in participation to decrease over time. Visual inspection of model forecasts and consultation with parkrun UK were used to confirm that this was a feasible counterfactual. To account for the zero weekly finishers during the pandemic period, a term $\lambda \beta _{3}$ was included in the model where $\lambda$ = 1 during the pandemic period, 0 otherwise. The immediate change in participation was included in the model as $\sigma \beta _{4}$ where $\sigma$ = 1 after parkruns reopened, 0 otherwise. The change in trend was included as $\beta _{5} T + \beta _{6} T^{2}$ where T is the number of weeks since the reopening parkrun events. Linear and quadratic terms were included to allow both the rate of growth and the change in the rate of growth over time to differ compared with before the pandemic. All models were adjusted for seasonality multiplicatively. Separate models were fit for each IMD quintile for ease of interpretability and to allow the pre- and post-pandemic trends and the post-pandemic step-change to differ by IMD. Model residuals were plotted to assess model fit. All analyses were undertaken in R version 4.3.1 (2023-06-16) [28].

## Results

### Descriptive statistics

**Fig. 1:**
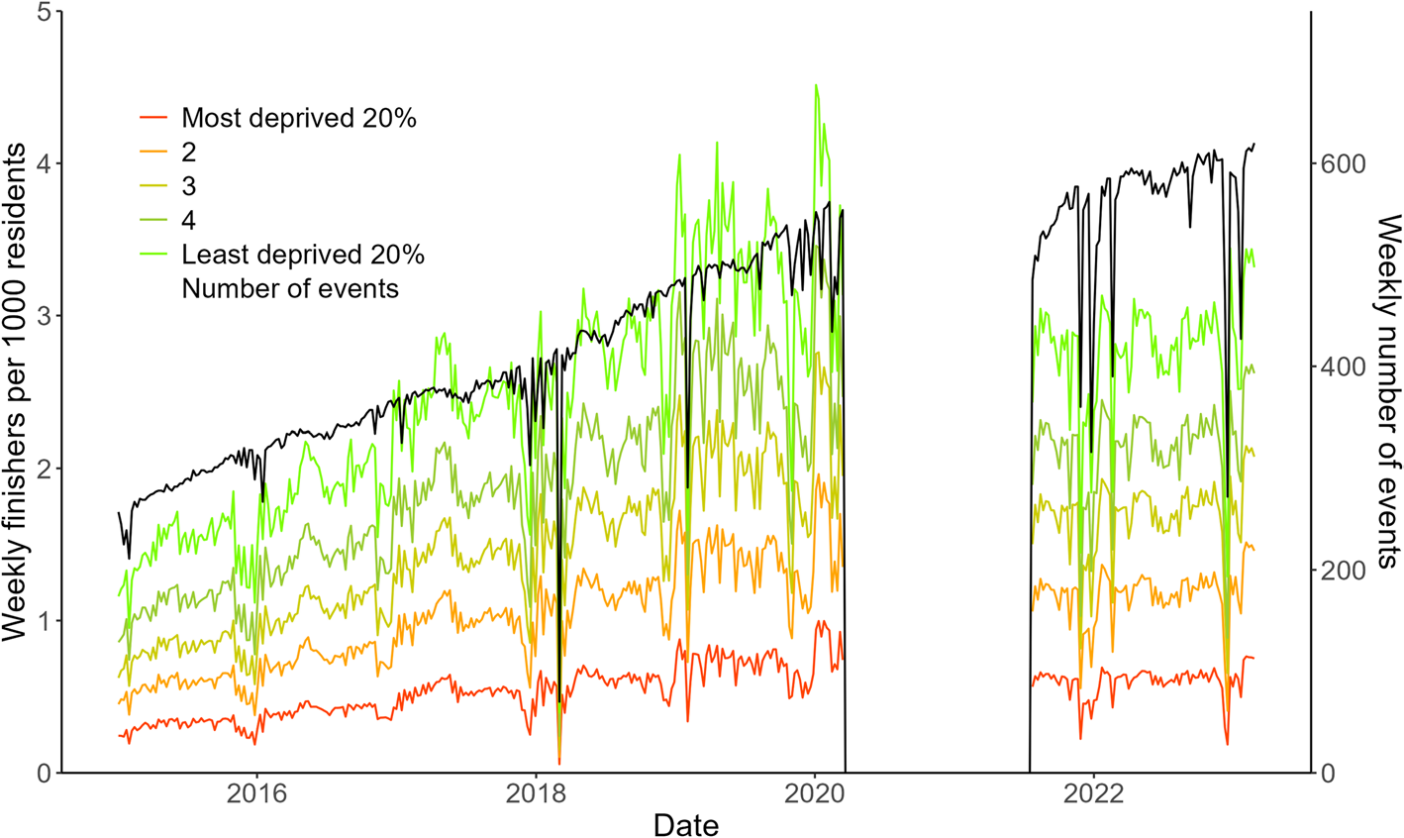
Weekly number of parkrun finishers in England per 1,000 residents by Index of Multiple Deprivation Quintile, and number of parkrun events in operation, from January 2015 to February 2023

Participation rates by IMD quintile (Y axis 1), and weekly number of events run (Y axis 2), are shown for the entire study period in Fig. 1. Participation rates increased near-linearly prior to the pandemic as did the number of events per week. The rates were ordered by IMD quintile, with more deprived communities having considerably lower rates of participation in parkrun than less deprived communities. Large temporary drops in the weekly number of events are explainable by event cancellations, usually due to adverse weather conditions. During the pandemic period from March 2020 to July 2021 no events were run and there was no participation. Participation in all IMD quintiles were visibly lower after the pandemic period compared to before. The number of weekly events continued to increase after the pandemic period.

### Interrupted time series

**Fig. 2:**
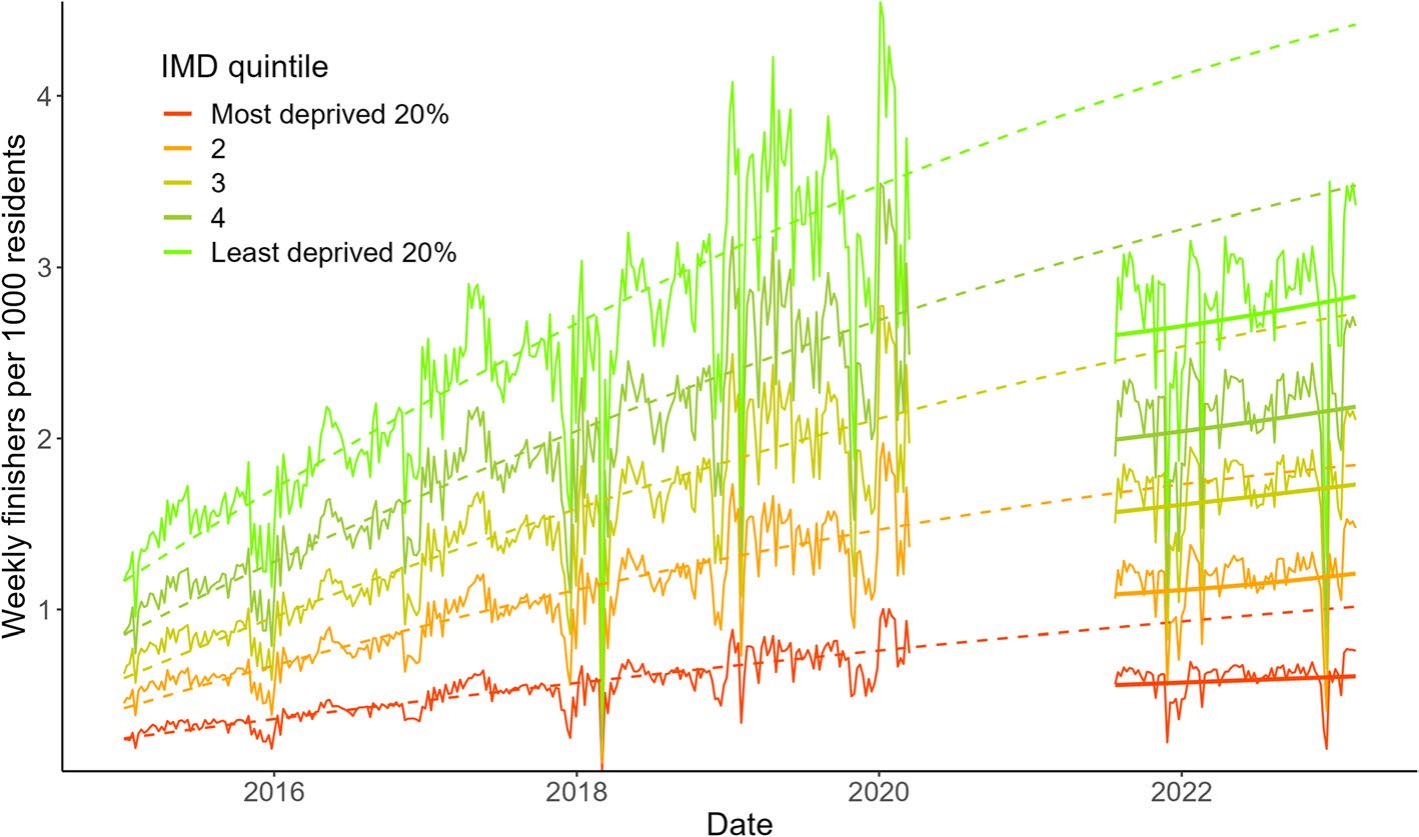
Counterfactual (expected participation in the absence of the pandemic) compared to observed participation. The thin lines show the observed participation rates by IMD each week. The dotted lines show the pre-pandemic trend ignoring seasonal variation extrapolated across the study period. The solid lines show the post-pandemic trend, ignoring seasonal variation

Figure 2 adds two lines to Fig. 1: the dotted line shows the pre-pandemic trend ignoring seasonal variation extrapolated across the study period, and the thick line shows the post-pandemic trend in weekly participation, ignoring seasonal variation. The difference between the dotted line and the solid line in August 2021 is a visual representation of our estimate of the step-change in parkrun participation. The difference between the slope of the dotted line and the slope of the solid line between July 2021 and February 2023 is a visual representation of the change in the rate of growth in participation after the return of parkrun. Appendix Fig. 3 shows observed compared to predicted participation rates including seasonality in the absence of the pandemic for each IMD quintile.

The segmented regression model for the total population (Table 2) showed that, before the pandemic, weekly parkrun participation increased by 34% per year (RR=1.34 [95% CI: 1.29, 1.40]). This rate of growth slowed by 2% per year (RR= 0.98 [95% CI: 0.98, 0.99]). The return of parkrun after the pandemic period was associated with an immediate decrease (step-change) in weekly participation of 29% (RR= 0.71 [95% CI: 0.63, 0.80]). Growth was 24% slower after the pandemic compared to before at (RR= 0.76 [95% CI: 0.61, 0.93]) however the rate of growth was 21% higher than before (RR= 1.21 [95% CI: 1.08, 1.36]) such that after the pandemic the growth in weekly participation every week was increasing over time. Based on the pre- and post-pandemic trends in weekly participation, weekly participation was expected to reach the same level as if the pandemic had not occurred in October 2023. The step change and change in trend of parkrun participation associated with the pandemic was similar in all IMD quintiles.

**Table 2:** Rate ratios from segmented poisson regression models. A separate model was fit for each IMD quintile and for the total population. IMD quintiles range from 1 (the most deprived 20%) to 5 (the least deprived 20%)

	Rate Ratio (RR) (95% Confidence Interval (CI))				
IMD quintile	Pre-pandemic trend:linear term (per year)	Pre-pandemic trend: quadratic term (per year)	Step change	Change in trend: linear term (per year)	Change in trend: quadratic term (per year)
1	1.32 (1.27-1.38)	0.99 (0.98-0.99)	0.69 (0.61-0.78)	0.74 (0.59-0.93)	1.21 (1.07-1.38)
2	1.37 (1.32-1.43)	0.98 (0.97-0.99)	0.71 (0.63-0.81)	0.76 (0.61-0.94)	1.22 (1.08-1.38)
3	1.37 (1.32-1.43)	0.98 (0.97-0.99)	0.70 (0.62-0.79)	0.77 (0.62-0.95)	1.20 (1.07-1.36)
4	1.34 (1.29-1.40)	0.98 (0.98-0.99)	0.70 (0.62-0.79)	0.76 (0.61-0.94)	1.21 (1.07-1.36)
5	1.32 (1.27-1.38)	0.98 (0.98-0.99)	0.71 (0.63-0.80)	0.76 (0.61-0.93)	1.21 (1.07-1.36)
Total	1.34 (1.29-1.40)	0.98 (0.98-0.99)	0.71 (0.63-0.80)	0.76 (0.61-0.93)	1.21 (1.08-1.36)

During the pandemic period (March 2020 - July 2021) no events took place and there were 8,955,818 fewer finishes than estimated in the absence of the pandemic. Between July 2021 (when parkrun returned) and February 2023, there were 3,801,326 fewer finishes than expected (Table 3). This equates to an average of 47,517 fewer parkrun finishes than expected per week since the return of parkrun. Overall, the model estimates that there were 12,757,144 total fewer parkrun finishes between March 2020 and February 2023 than would have occurred in the absence of the pandemic. See Appendix Table 4 for the absolute predicted and observed finishes split by IMD.

**Table 3:** The number of finishes according to the counterfactual models (what we expect would have happened in the absence of the pandemic); the observed finishes; and the difference between these. Estimated for two time periods

	Parkrun finishes		
Time period	Counterfactual N	Observed N	Difference N (% decrease)
March 2020-July 2021	8,955,818	0	8,955,818 (100)
July 2021-February 2023	11,456,767	7,655,441	3,801,326 (32.7)
**Total (March 2020-February 2023)**	**20,412,585**	**7,753,272**	**12,757,144 (62.5)**

## Discussion

### Key findings

The pandemic was shown to be associated with an immediate decrease in parkrun participation for all IMD quintiles upon reopening of parkrun events. Growth in participation was initially lower after reopening compared to before the pandemic. However, this rate of growth was shown to be increasing over time. In contrast to our hypothesis, the relative impact of the pandemic on parkrun participation was similar for all IMD quintiles such that relative differences in parkrun participation by IMD remained unchanged compared with the pre-pandemic period. Given that the weekly number of events continued to rise after parkrun returned, the lasting effect of the pandemic is unlikely to be attributable to geographical reductions in access to parkrun events.

### Comparison with wider literature and implications of findings

The reduction in parkrun participation is in accordance with the wider decrease in running activity in the UK since 2021 and may contribute to said trends [23]. However, running activity in the UK has been decreasing since 2016 [23] whilst parkrun participation was increasing prior to the pandemic. Our findings are also in agreement with previous analysis of specific Scottish parkrun events which found a 13% reduction in participation in the year after parkrun returned compared to the year before the pandemic but no significant difference in this effect by the deprivation of the parkrun location [7].

In contrast to previous findings regarding physical activity levels [23] and our original hypothesis, our study did not find a clear socioeconomic impact of the pandemic break on participation in the return to parkrun. Instead, the inequalities in participation according to IMD continue as before. This disparity could be explained by parkrun’s targeted opening of new events in locations that improve access for more deprived communities [10, 18]. Our findings suggest that more work is needed to increase parkrun participation, particularly of those from the most deprived communities. Indeed, improving parkrun participation could provide a high social return on investment through its wellbeing benefits [25].

This study looked only at parkrun participation, not wider physical activity levels. It is possible that the reduction in parkrun participation after the pandemic is in part due to a shift from organised community-level activity such as parkrun toward other types of physical activity. Indeed, Sport England data [23] showed that there were a similar proportion of adults who were physically active in 2021-2022 compared to before the pandemic and that more individuals taking part in team sports and walking for leisure in 2021-2022 compared to before the pandemic.

Whilst we cannot rule out parkrun participants replacing parkrun with other forms of physical activity after the pandemic, the reduction in parkrun participation has implications for the wellbeing of would-be participants and for the local communities which previously benefited from improvements in social capital brought about by parkrun events.

### Strengths

This study utilises a comprehensive dataset provided by parkrun, with participation data spanning across 32,470 Lower Layer Super Output Areas (LSOA) and over 686 weeks from 2010 to 2023. The granularity of this data empowers us to draw meaningful insights from descriptive statistics alone and the length of the study period before and after the pandemic period reduces uncertainty in the interrupted time series analysis. The size of this dataset allowed for more precise estimates of the effect of the pandemic than previous analyses which focused on a limited number of parkrun events at the event level [7]. Use of IMD according to the LSOA of one’s home address gives a more accurate estimate of the effect of the pandemic by socioeconomic status than one based on event location, since many people travel to events which may be in areas with different socioeconomic profiles than the area in which they live. This is particularly the case in cities where there are pockets of deprivation located close to parkruns held in affluent areas [8]. The use of interrupted time series analysis allowed for an estimation of both the immediate and long-term impacts of the pandemic on parkrun participation accounting for seasonal variation and pre-pandemic trends.

### Limitations

Given the widespread impact of the pandemic, there is no suitable control group for analyses. Instead, we rely on a counterfactual derived from extrapolating participation trends observed from 2015 to 2019. As such, we are unable to account for secular changes during the study period such as the rising cost of living [6]. This limits our ability to infer causality. The length of the hiatus, spanning 14 months, introduces uncertainty to effect estimates resulting from assumptions about the rate of growth. The data provided by parkrun for this study was aggregated at the LSOA level. This prevents conclusions being drawn at the individual level and precludes analysis of the effect of individual characteristics, such as gender, age, and finish-time, on participation. It is possible that a small number of parkrun participants forget to scan their parkrun barcode after completing a parkrun event with these finishes therefore not recorded in our dataset. However, we do have data for a very large number of participants so do not expect these missing data to change our findings. LSOA population and IMD were assumed to be constant throughout the study period. In reality, these metrics would have changed slightly over the study period. However, these changes are generally small [12] and the effect of this on our findings is likely to be minimal. We were unable to assess physical activity levels of those who reduced or stopped their parkrun participation during this period. As such, we cannot know if reduced parkrun participation will have resulted in reduced physical activity levels. Finally, this study cannot isolate the effects of specific pandemic policies or of the pandemic itself. As such, we refrain from offering recommendations for future pandemic responses. Nevertheless, we aspire for this analysis to contribute to a broader conversation about the comparative costs and benefits of organised outdoor recreation amid a communicable disease pandemic.

## Conclusion

parkrun was not immune to the pandemic. The overall impact of the pandemic and related policy on parkrun participation was stark, with a significant and prolonged decrease in participation rates in all quintiles of socioeconomic deprivation. The targeted opening of new parkrun events in more deprived areas may have mitigated differences in the impact of the pandemic on parkrun participation between different groups in society. However, socioeconomic inequalities in participation remain as before, and more work is required to increase parkrun participation, particularly in the most deprived communities.

We cannot rule out individuals replacing parkrun with other forms of physical activity. However, this reduction in participation likely had a negative impact on the wellbeing of would-be parkrun participants and the social capital of their communities. Policymakers may want to consider the trade-off between the short-term benefits of reduction in transmission of an infectious disease in outdoor physical activity settings, and the negative consequences of a prolonged reduction in community based physical activity.

## Figures and Tables

**Fig. 3:**
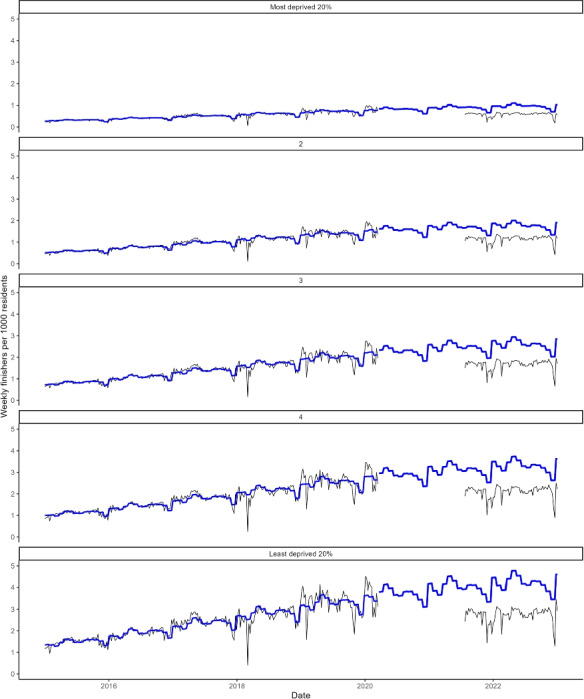
The black line shows the observed Weekly number of parkrun finishers in England per 1,000 residents by Index of Multiple Deprivation Quintile from January 2015 to February 2023. The blue line shows the predicted participation rates in the absence of the pandemic for the same period

## Data Availability

All aggregated parkrun data is provided open-source, alongside R code, at https://github.com/RobertASmith/parkrun_temporal_23/tree/v1.0 to enable replication.

## Appendix

**Table 4:** The number of finishes according to the counterfactual models (what we expect would have happened in the absence of the pandemic); the observed finishes; and the difference between these. Estimated for two time periods. Split by IMD quintile

	Parkrun finishes from July 2021 to February 2023			Parkrun finishes from March 2020 to February 2023		
IMD quintile	Counterfactual N	Observed N	Difference N, (% decrease)	Counterfactual N	Observed N	Difference N, (% decrease)
5 (Least deprived 20%)	3,694,476	2,492,779	1,201,697 (32.5)	6,585,536	2,492,779	4,092,757 (62.1)
4	2,928,999	1,942,000	986,999 (33.7)	5,209,122	1,942,000	3,267,122 (62.7)
3	2,358,482	1,570,394	788,088 (33.4)	4,200,953	1,570,394	2,630,559 (62.6)
2	1,618,256	1,100,078	518,178 (32.0)	2,902,574	1,100,078	1,802,496 (62.1)
1 (Most deprived 20%)	856,554	550,190	306,364 (35.8)	1,514,400	550,190	964,210 (63.7)

## Abbreviations

IMD: Index of Multiple Deprivation
LSOA: Lower Super Output Area
RR: Rate Ratio
CI: Confidence Interval
